# Effect of silencing *Thrips palmi Btk29A* and *COL3A1* on fitness and virus acquisition

**DOI:** 10.3389/fmicb.2023.1254246

**Published:** 2023-10-19

**Authors:** Vavilapalli Rajesh, Sumit Jangra, Amalendu Ghosh

**Affiliations:** ^1^Insect Vector Laboratory, Advanced Centre for Plant Virology, Indian Agricultural Research Institute, New Delhi, India; ^2^Division of Entomology, Indian Agricultural Research Institute, New Delhi, India

**Keywords:** melon thrips, GBNV, groundnut bud necrosis orthotospovirus, RNAi, dsRNA, virus-vector relationship

## Abstract

*Thrips palmi* (Thysanoptera: Thripidae) is a major agricultural pest infesting over 200 plant species. Along with direct injury caused by feeding, *T. palmi* spreads several orthotospoviruses. *Groundnut bud necrosis orthotospovirus* (GBNV, family *Tospoviridae*, genus *Orthotospovirus*) is the predominant orthotospovirus in Asia, vectored by *T. palmi*. It is responsible for almost 89 million USD losses in Asia annually. Several transcripts of *T. palmi* related to innate immune response, receptor binding, cell signaling, cellular trafficking, viral replication, and apoptosis are responsive to the infection of orthotospoviruses in thrips. Expression of *T. palmi tyrosine kinase Btk29A isoform X1* (*Btk29A*) and *collagen alpha-1(III) chain-like* (*COL3A1*) are significantly regulated post-GBNV and *capsicum chlorosis orthotospovirus* infection. In the present study, *T. palmi Btk29A* and *COL3A1* were silenced and the effect on virus titer and fitness was assessed. The expression of *Btk29A* and *COL3A1* was significantly reduced by 3.62 and 3.15-fold, respectively, 24 h post-dsRNA exposure. Oral administration of *Btk29A* and *COL3A1* dsRNAs induced 60 and 50.9% mortality in *T. palmi*. The GBNV concentration in *T. palmi* significantly dropped post-silencing *Btk29A*. In contrast, the silencing of *COL3A1* led to an increase in GBNV concentration in *T. palmi* compared to the untreated control. To the best of our knowledge, this is the first report on the effect of silencing *Btk29A* and *COL3A1* on the fitness and GBNV titer in *T. palmi.*

## Introduction

The melon thrips, *Thrips palmi* Karny (Thysanoptera: Thripidae), is a major pest of vegetables, legumes, and ornamental plants. *T. palmi* was first identified in Sumatra (Karny, 1925) and is currently reported from the rest of Asia, Australia, the Pacific, North and South America, and West Africa (Cannon et al., 2007). It is considered an A1 quarantine pest by EPPO [The European and Mediterranean Plant Protection Organization (EPPO), 2018]. Larvae and adults of *T. palmi* rasp the tender plant parts and suck the cellular content. White silvery scarring develops on the affected plant parts that gradually turn brownish. In addition to direct injury caused by feeding, *T. palmi* transmits many orthotospoviruses (Riley et al., 2011). *T. palmi*-transmitted groundnut bud necrosis orthotospovirus (GBNV) causes more than USD 80 million losses annually in Asia (Reddy et al., 1995). GBNV can cause yield losses of up to 100% in tomatoes (Venkata Ramana et al., 2011). Outbreaks of watermelon bud necrosis orthotospovirus (WBNV) transmitted by *T. palmi* resulted in the failure of watermelon cultivation in southern India (Singh and Krishnareddy, 1995). The economic losses caused by *T. palmi* during 1994–2004 were around £ 16.9–19.6 million (MacLeod et al., 2004), though the exact figures on worldwide economic impact are not available. The application of insecticides is the most common option to protect crops from thrips and orthotospoviruses. However, thrips develop quick resistance to most of the common classes of insecticides due to their high fecundity, short generation period, and wide host range (Bao and Sonoda, 2012; Bao et al., 2014; Shi et al., 2020, 2021).

Lately, advancements in genome-assisted pest management techniques paved the way for inventive pest control methods. In particular, RNA interference (RNAi) has been extensively used to control chewing pests by delivering dsRNA through various techniques (Yu et al., 2013; Mamta and Rajam, 2017; Vogel et al., 2019; Adeyinka et al., 2020; Christiaens et al., 2020; Zhu and Palli, 2020; Hough et al., 2022). However, limited knowledge of the gene functions of target pests restrains the scope of genome-assisted insect resistance programs. RNAi has been successfully utilized to study the gene function of *Frankliniella occidentalis* and *T. tabaci* (Badillo-Vargas et al., 2015; Han et al., 2019; Singh et al., 2019; Andongma et al., 2020; Wu et al., 2022; Zhang et al., 2022a,b). Silencing of *hsp70, TLR3,* and *TOB1* induced significant mortality and also inhibited chili leaf curl virus (ChiLCV) transmission by *Bemisia tabaci* adults (Chakraborty and Ghosh, 2022; Thesnim et al., 2023). *Aphb* and *CP19* silencing affected fitness of aphid species (Mao and Zeng, 2012; Shang et al., 2020). RNAi of *Nilaparvata lugens Nldl* and *Nljag* in nymphs produced lethal or teratogenic effects (Yang et al., 2022). Little attempts were made to study the gene functions of *T. palmi*. In our previous study, *T. palmi* transcripts related to innate immune response, receptor binding, cell signaling, cellular trafficking, viral replication, and apoptosis were responsive to the acquisition of GBNV (Mahanta et al., 2022). Silencing of *T. palmi UHRF1BP1* and *PFAS* using modified anti-sense oligos showed a decline in GBNV titer in *T. palmi* (Priti et al., 2022). Besides, *T. palmi tyrosine kinase Btk29A isoform X1* (*Btk29A*) and *collagen alpha-1(III) chain-like* (*COL3A1*) were significantly regulated in response to GBNV and capsicum chlorosis orthotospovirus (CaCV) infection (Widana Gamage et al., 2018; Mahanta et al., 2022). Btk29A is involved in ATP binding and cellularization of *Drosophila* embryos (Thomas and Wieschaus, 2004). Dysfunctional *Btk* causes selective apoptosis and decreases HIV-1 production in HLM-1 cells (Guendel et al., 2015) indicating that *Btk* positively regulates productive virus infection either by activating the infected cells or enhancing the release of the virus from the infected cells. Collagens are structural proteins of the extracellular matrix and basal membrane and are constituents of viral biofilm (VB) development of human T-cell leukemia virus type 1 (HTLV-1; Millen et al., 2019). HTLV-1 infects T- cells through cell-to-cell transmission by polarized budding into synaptic clefts and cell surface transfer of VBs. Knockout of collagen impairs the transfer of viral protein from infected to acceptor cells. Type III collagen is also involved in cell adhesion, migration, and differentiation through its interaction with cell surface receptor integrins (Kim et al., 2005). In the present study, *T. palmi Btk29A* and *COL3A1* were silenced to study the effect on thrips fitness and orthotospovirus titer in thrips.

## Materials and methods

### Homogenous population of *Thrips palmi*

The study employed a homogenous population of *T. palmi* derived from a single adult female. The isofemale line has been maintained on eggplant since 2018. The identity of the population was further verified by morphometric keys (Bhatti, 1980; Cluever and Smith, 2017) and mitochondrial cytochrome oxidase subunit I (*mtCOI*) nucleotide sequencing.

### Groundnut bud necrosis orthotospovirus culture

The initial GBNV inoculum was collected from a pure culture maintained at the Advanced Center for Plant Virology, Indian Agricultural Research Institute (IARI), New Delhi. Sap inoculation of healthy cowpea plants at the two-leaf stage was done with GBNV under insect-proof conditions as described by Ghosh et al. (2021). The presence of GBNV infection in cowpea was verified using RT-PCR using GBNV-specific primers AG109F-AG110R (Table 1).

**Table 1:** List of primers used in the study.

S. No.	Gene name	Primer Name	Primer Sequence (5′ → 3′)	Annealing temperature (°C) in PCR	Amplicon size (bp)	Melting temperature (°C) in RT-qPCR	Purpose	References
1	MtCoI	LCO1490	GGTCAACAAATCATAAAGATATTGG	50	657	–	*Thrips palmi* identification	Folmer et al. (1994)
		HCO2198	TAAACTTCAGGGTGACCAAAAAATCA					
2	GBNV M segment	AG109F	CCATCTACTTCAGTAGAAAACACTAG	59	1767	–	Diagnosis of GBNV	Mahanta et al. (2022)
		AG110R	AGAGCAATCAGTGCAACAATTAAATA					
3	*β- tubulin*	AG171F	CCAGCCACATTCCTGGATAC	55	117	79.5	Endogenous control gene	Widana Gamage et al. (2018)
		AG172R	ATGCGTTGGCAGTCACATAC					
4	*Tyrosine-protein kinase Btk29A isoform x1*	AG507F	CTGAAGAATACGGAAGTCGT	55	279	86.6	dsRNA synthesis, RT-qPCR	This study
		AG508R	TGAGCAGAAAGAGTCAATCG					
5	*Collagen alpha-1(III) chain-like*	AG198F	AAAACTGCAGGTGGAAATGCCTCAAACGCA	55	257	81.1	dsRNA synthesis, RT-qPCR	This study
		AG199R	AAAATCTAGATGAGGAGTCAGGAGGATCACA					
6	*Bemisia tabaci transducer of erbB2.1*	AG301F	AGGTCAGCTATAGGATTGGT	53	167 bp	–	dsRNA synthesis	Thesnim et al. (2023)
		AG302R	TGAGCTGACTTAAACTGGAC					
7	GBNV *nucleocapsid protein (N)*	AG335F	CTGGTGGCTCTGCAGATG	54	219	80	Estimation of GBNV copies	Priti et al. (2022)
		AG336R	CATCTGGCCCTACGTCAG					

### Designing of dsRNA constructs

*T. palmi* differential gene expression data in response to GBNV and CaCV (Widana Gamage et al., 2018; Mahanta et al., 2022) were analyzed for the selection of potential gene targets in response to orthotospovirus infection. The expression of the *Btk29A* and *COL3A1* in *T. palmi* was highly abundant post-GBNV and CaCV acquisition. Therefore, *T. palmi Btk29A* and *COL3A1* were targeted for silencing using the cognate dsRNA. The available sequences of *Btk29A* and *COL3A1* were retrieved from NCBI and aligned in MEGAX (Kumar et al., 2018) to find the conserved regions. SiRNA Wizard web tool (accessed on 10-01-2022)^1^ was used to predict putative siRNAs from the conserved regions of *T. palmi Btk29A* and *COL3A1*. The region identified for dsRNA design was subjected to evaluation for off-target effects in humans, mice, birds, butterflies, bees, ants, and plants using the NCBI BLASTn tool.^2^ The region in both genes that showed the maximum number of putative siRNAs without having any off-target effect was selected for dsRNA synthesis. The selected regions were 1,166–1,444 for *Btk29A* and 213–469 for *COL3A1* genes.

### Synthesis of dsRNA constructs

The dsRNA stretches were amplified using primer pairs listed in Table 1. The PCR conditions were optimized using a gradient PCR. Total DNA was isolated from *T. palmi* using a CTAB extraction buffer as described by Jangra and Ghosh (2022) and used for amplification in a thermal cycler. A 25 μL PCR mixture comprised of 1X PCR buffer (Thermo Fisher Scientific, United States), 0.4 μM each forward and reverse primer (GCC Biotech, India), 0.26 mM dNTP mix (Thermo Fisher Scientific), ~ 60 ng template DNA, and 2 U of DreamTaq DNA polymerase (Thermo Fisher Scientific) were used. PCR was performed in a T100 thermocycler (Bio-Rad, United States) with an initial denaturation at 94°C for 5 min, 35 cycles of denaturation at 94°C for 40 s, annealing at 55°C for 40 s, extension at 72°C for 40 s, followed by a final extension at 72°C for 10 min. The PCR products were resolved on a 2% agarose gel, stained with GoodView (BR Biochem, India), and visualized under a gel documentation system (MaestroGen Inc., Taiwan). The amplified PCR products were sequenced for confirmation purposes.

The PCR amplicons were cloned between two T7 promoters in the L4440 expression vector (Addgene, United States) and transformed into RNase III deficient *E. coli* HT115 cells (University of Minnesota, United States). The presence of the desired inserts was confirmed by colony PCR and restriction digestion. Production of dsRNA was induced by adding 0.8 M isopropyl-β-D-1-thiogalactopyranoside (IPTG, HiMedia, India) to the culture of recombinant *E. coli* HT115 cells and incubating overnight at 37°C in a shaking incubator (Gnanasekaran et al., 2019, 2021). Total RNA was extracted from HT115 cells using Trizol reagent (Invitrogen, United States) according to the manufacturer’s protocol. DsRNA from the total RNA was purified using 1 U of DNase I (RNase-free, Thermo Fisher Scientific) and 1 U of RNase A (DNase and protease-free, Thermo Fisher Scientific) for 1 h at 37°C in the presence of 500 mM sodium chloride. The enzymes were inactivated by chloroform extraction as described by Chakraborty and Ghosh (2022). The quality and concentration of the purified dsRNAs were assessed in a spectrophotometer (Nano-300, Genetix Biotech Asia, India) and visualized on a 2% agarose gel. A dsRNA targeting *Bemisia tabaci transducer of erbB2.1* (*BtTOB1*; Thesnim et al., 2023) and not specific to *T. palmi* based on nucleotide homology screening was used as a negative control.

### Delivery of *Btk29A* and *COL3A1* dsRNAs to *Thrips palmi*

The purified *Btk29A* and *COL3A1* dsRNAs were administered to *T. palmi* orally using an artificial feeding setup as reported by Priti et al. (2022). Briefly, a diet was prepared by mixing 50 mg/mL pine pollen (Lost Empire Herbs, United States) extract with 1% sucrose solution and 0.0001% methylene blue tracker dye (Bio Basic, United States). The diet was supplemented with 3 μg/μL dsRNA (total volume 300 μL). The diet with dsRNA was filled into UV-sterilized detachable caps of 2 mL microcentrifuge tubes and covered with stretched parafilm. Around 20 adults or larvae were taken in the perforated microcentrifuge tubes. The cap filled with diet was closed gently. *T. palmi* were allowed to feed for 24 h at 28 ± 1°C temperature, 60 ± 10% relative humidity, and 16 h light–8 h dark. In the control sets, a diet without dsRNA and a diet with *BtTOB1* dsRNA (non-specific to *T. palmi*) were supplied. Several such replicates were maintained to get enough dsRNA-treated and untreated thrips to assess the mortality, expression of the target genes, and virus titer in thrips.

### Effect of *Btk29A* and *COL3A1* dsRNAs on cognate mRNA expression

The expression of *T. palmi Btk29A* and *COL3A1* was assessed 24 h post-dsRNA feeding by RT-qPCR. *β-tubulin* was taken as the endogenous control. Table 1 lists the primer pairs used in RT-qPCR. Five surviving *T. palmi* in three biological replicates for each gene under study were considered for estimating the mRNA expression level. Total RNA was extracted using NucleoSpin RNA XS (Macherey-Nagel, Germany) according to the manufacturer’s protocol. Total RNA was quantified in a Nano-300 Micro Spectrophotometer (Genetix Biotech Asia), and cDNA was synthesized using FIREScript RT cDNA synthesis kit (Solis BioDyne, Tartu, Estonia). One μg RNA template, 5 μM oligo dT primers, 500 μM dNTP mix, 2 μL of 1 X reaction buffer, 10 U FIREScript RT, and 1 U RiboGrip RNase inhibitor were used in 20 μL reaction mixture. cDNA was synthesized in a T100 thermocycler (BioRad, United States) by reverse transcription at 50°C for 60 min, and enzyme inactivation at 85°C for 5 min. The qPCR assay was performed in an Insta Q48M real-time PCR (Himedia, India). The 20 μL qPCR mixture included 1X GoTaq qPCR Master Mix (Promega, United States), 300 nM CXR passive reference dye, 0.25 μM of each forward and reverse primer, and 2 μL of template cDNA. The following thermal cycling was followed: initial denaturation at 94°C for 5 min, 40 cycles of 94°C for 40 s, 56°C for 40 s, and 72°C for 40 s. A melting curve analysis was carried out after each reaction to assess the specificity of the amplicons. Three biological and two technical replicates were used in the RT-qPCR. A non-template reaction is used as a negative control. The relative expression of *Btk29A* and *COL3A1* in dsRNA-fed *T. palmi* was measured following the 2^−ΔΔCT^ method (Livak and Schmittgen, 2001). The statistical analysis was performed and graphs were generated in Microsoft Excel 2019. Expression of *T. palmi Btk29A* and *COL3A1* in *BtTOB1* dsRNA-fed thrips served as the negative control.

### Effect of *Btk29A* and *COL3A1* dsRNAs on survival of *Thrips palmi*

*T. palmi* individuals that fed on the artificial diet were identified by a blue tinge in their abdomen and were exclusively considered to assess the effect of dsRNA on survival. The mortality of *T. palmi* was recorded 24 h post-dsRNA feeding. The whole experiment was replicated nine times for each treatment. The mean mortality in dsRNA-exposed *T. palmi* was recorded and compared with the thrips fed on the dsRNA-free diet and diet with *BtTOB1* dsRNA. Tukey’s test was performed with XLSTAT 2014.5.03 to differentiate means across categories with a 95% confidence interval. The morphological changes, if any, post-dsRNA feeding were also checked under a stereo microscope (M205 FA, Leica, Germany) and captured in a Leica DFC425 C.

### Effect of *Btk29A* and *COL3A1* dsRNAs on groundnut bud necrosis orthotospovirus titer in *Thrips palmi* larvae

The eggs of *T. palmi* were collected using the artificial oviposition setup reported by Jangra et al. (2020). The eggs were incubated at 28°C on wet tissue paper. The newly hatched first instar larvae (L1) were collected using a Camel hairbrush and allowed to feed on a diet mixed with dsRNA for 2 h as outlined above. *T. palmi* fed on the dsRNA-free diet and diet with *BtTOB1* dsRNA were used as negative controls. The dsRNA-fed and non-fed L1 were allowed to acquire GBNV as reported by Ghosh et al. (2021). In brief, *T. palmi* L1s were released for a 12-h acquisition access period (AAP) on detached GBNV-infected cowpea leaf at 28 ± 1°C and 60 ± 10% relative humidity. Five live L1s at three biological replicates were collected in a microcentrifuge tube post-GBNV acquisition and used to quantify the virus copies. The GBNV titer acquired by dsRNA-fed and non-fed *T. palmi* larvae were estimated by absolute quantification by RT-qPCR using the primer pair AG335F-AG336R (Table 1). Total RNA extraction and cDNA synthesis was performed as outlined above. One μg RNA template, 5 μM random primers, 500 μM dNTP mix, 2 μL of 1 X reaction buffer, 10 U FIREScript RT, and 1 U RiboGrip RNase inhibitor were used in 20 μL reaction mixture. The cDNA was synthesized in a T100 thermal cycler with primer annealing at 25°C for 10 min, reverse transcription at 50°C for 60 min and enzyme inactivation at 85°C for 5 min. 10 μL of 1X GoTaq qPCR Master Mix (Promega), 300 nM CRX reference dye, 0.25 μM of each forward and reverse primer, and 2 μL template cDNA were used in 20 μL qPCR reaction mixture. Thermal cycling was performed as initial denaturation at 95°C for 5 min, 35 cycles at 95°C for 25 s, 54°C for 25 s, and 72°C for 30 s. A melting curve analysis was performed to ensure the reaction’s specificity. Three biological and two technical replicates were used for each treatment. A standard curve (y = −3.3251x + 28.141, efficiency = 99.86%) of GBNV using primer pair AG335F-AG336R reported by Priti et al. (2022) was used for the quantification of GBNV copies in *T. palmi*.

The viral titer was estimated by fitting the mean CT values in the standard curve. The following formula was used to calculate the virus copy number in Microsoft Excel 2019. N = (x × 6.022 × 10^23^)/ (n × 340 × 10^9^), where N = number of viral copies, x = amount of amplicon in ng, and n = length of linearized plasmid.

One-Way ANOVA was performed to determine the mean differences in virus copies among treatments at *p* ≤ 0.05 in XLSTAT 2014.5.03. The effect of *Btk29A* and *COL3A1* dsRNAs feeding on survival and mRNA expression at this stage (L1) was also recorded as mentioned above.

## Results

### *Thrips palmi* population and groundnut bud necrosis orthotospovirus culture

*T. palmi* adults had quadrangular heads with seven segmented antennae. At the top of the head, three brick-red ocelli were present in a triangular formation. A pair of interocellar setae originated outside the ocellar triangle. Besides, the nucleotide sequence of a 595 bp portion of *mtCOI* was amplified using primer pairs LCO 1490 and HCO 2198 (Folmer et al., 1994) and was 100% identical with *T. palmi*. The sequence can be retrieved from GenBank with the accession number OP223495.

RT-PCR with GBNV-specific primers produced ~1767 bp desired amplicon from all the sap-inoculated plants. The nucleotide sequence was found to be more than 97% identical to other GBNV isolates. The sequence can be retrieved from GenBank with the accession number MN566913.

### Designing of dsRNA constructs and dsRNA synthesis

**Figure 1:**
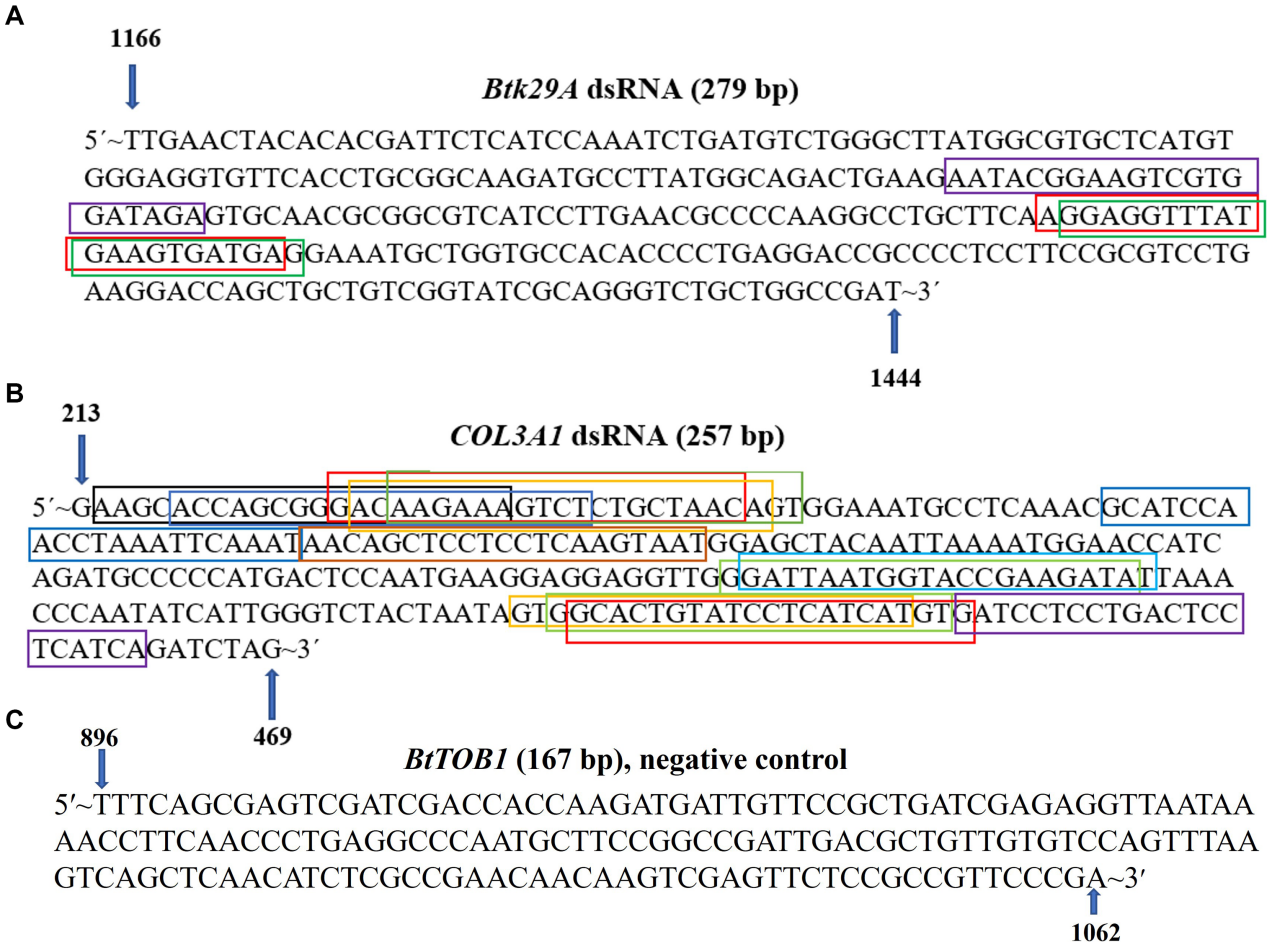
Design of *Btk29A* and *COL3A1* dsRNA. **(A)** A 279 nt conserved region of *Thrips palmi Btk29A* was selected for designing *Btk29A* dsRNA. **(B)** A 257 nt conserved region of *T. palmi COL3A1* was selected for designing *COL3A1* dsRNA. The putative siRNAs were marked in different colored boxes. **(C)** A 167 nt conserved region of *Bemisia tabaci TOB1,* not specific to *T. palmi* was used to design the negative control dsRNA.

A conserved 279 nt-long (1,166 to 1,444) fragment of the *Btk29A* gene (1.538 nt) and a 257 nt-long (213 to 469 nt) fragment of the *COL3A1* gene (7.063 nt) of *T. palmi* were preferred for dsRNA designing (Figure 1). The dsRNA stretches were unique to *T. palmi* and showed no homology with other non-target organisms such as *Homo sapiens* (taxid:9606), mice (taxid:10088), Lepidoptera (taxid:7088), *Aves* (taxid:8782), Hymenoptera (taxid:7399), Formicidae (taxid:36668), and plants (taxid:3193) in BLASTn analysis. In siRNA Wizard, the 279 nt-long *T. palmi Btk29A* fragment produced 3 putative siRNAs of 21 nt-long. Similarly, the 257 nt-long *COL3A1* fragment predicted 13 putative siRNAs of 21 nt-long (Figure 1). A conserved 167 nt (896–1,062) stretch of *B. tabaci TOB1* was used to synthesize *BtTOB1* dsRNA and considered as a negative control. The *BtTOB1* dsRNA was specific to *B. tabaci* (Thesnim et al., 2023) and showed no homology to *T. palmi*.

**Figure 2:**
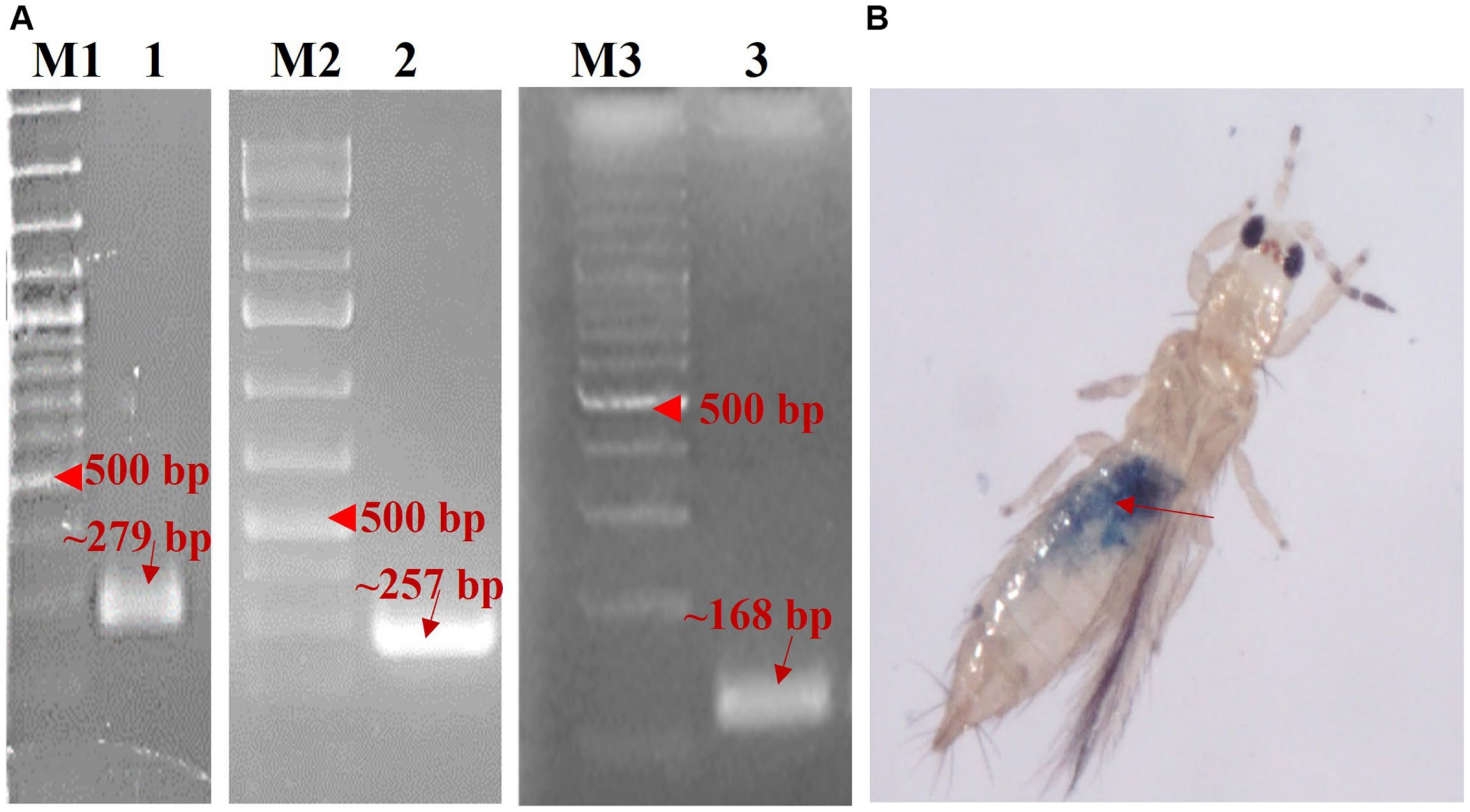
Synthesis and delivery of dsRNA. **(A)** Purified *Btk29A* dsRNA (1) and *COL3A1* dsRNA (2), *BtTOB1* dsRNA (3) used as a negative control, M1, M3: 100 bp plus DNA ladder, M2: 1 kb plus DNA ladder. **(B)** Adult *T. palmi* fed on artificial diet for 24 h indicated by blue tinge on abdomen (red arrow).

PCR with primer pair AG507F-AG508R specific to *Btk29A* yielded an amplicon of 279 bp (accession no. OP346052). Whereas a 257 bp (accession no. OP345816) fragment of *COL3A1* was amplified in PCR with the primer pair AG188F-AG189R. On a 2% agarose gel, the *Btk29A* and *COL3A1* dsRNAs purified from the total RNA of recombinant *E. coli* HT115 cells generated ~279 and ~ 257 bp bands, respectively (Figure 2). The concentration of the purified dsRNA was 621.5 ng/ μL for *Btk29A* and 531.0 ng/ μL for *COL31A*. It was 779.9 ng/μL in the case of *BtTOB1* dsRNA.

### Effect of *Btk29A* and *COL3A1* dsRNAs on mRNA expression

**Figure 3:**
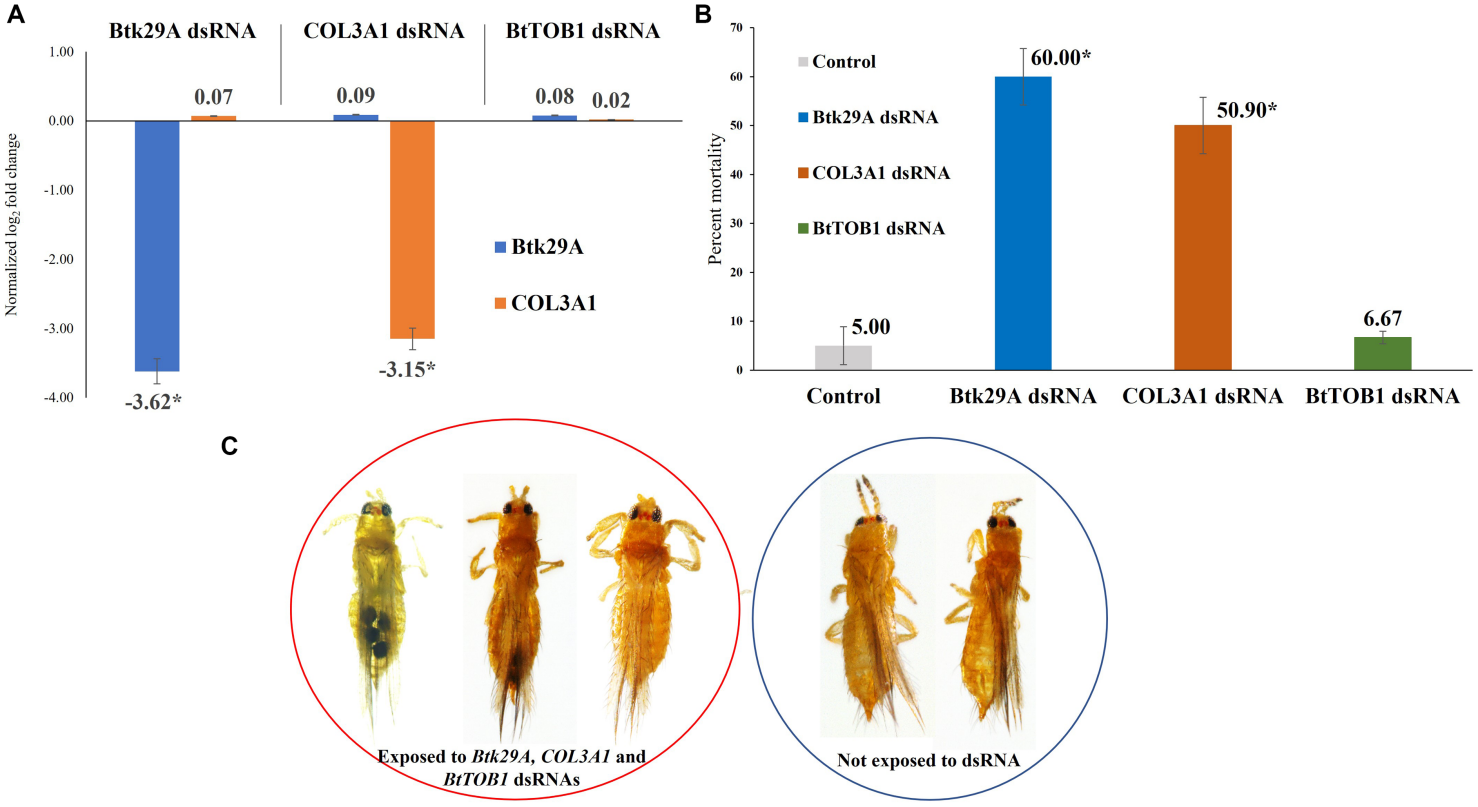
Effect of silencing *Btk29A* and *COL3A1* on survival of *T. palmi*. **(A)** Normalized relative expression of *T. palmi Btk29A* and *COL3A1* mRNA post-dsRNA exposure. **(B)** Percent mortality of adult *T. palmi* 24 h post-*Btk29A* and *COL3A1* dsRNA feeding at 3 μg/mL. The error bars are the standard error of the mean (SEM). Mean denoted by an asterisk (*) indicates a significant difference (*p* < 0.05). **(C)** *Btk29A* and *COL3A1* dsRNA-exposed and unexposed *T. palmi*. No morphological deformities were recorded post-dsRNA exposure.

The expression of *T. palmi Btk29A* and *COL3A1* was downregulated post-dsRNA feeding. The mean logarithmic expression of *T. palmi Btk29A* was estimated as −5.66 in the larval stage and 0.13 in the adult stage under controlled conditions. It was 2.53 and 0.39 in larval and adult stages, respectively for *T. palmi COL3A1*. A 3.62-fold downregulation of *T. palmi Btk29A* mRNA was recorded 24 h post-feeding on a diet containing *Btk29A* dsRNA in contrast to *T. palmi* feeding on a diet without dsRNA (Figure 3). Similarly, a 3.15-fold reduction in *T. palmi COL3A1* mRNA expression level was recorded in *T. palmi* fed on *COL3A1* dsRNA. Expression of *T. palmi Btk29A* and *COL3A1* post-exposure to *BtTOB1* dsRNA was statistically non-significant with untreated control. There was no significant difference in the expression of the endogenous control, *β-tubulin*, between dsRNA-fed and non-fed *T. palmi*, indicating that the *Btk29A* and *COL3A1* *dsRNAs* had a specific action on the target mRNAs. The primer pairs for *Btk29A*, *COL3A1*, and *β-tubulin* produced no secondary peaks in the RT-qPCR melting curve analysis, indicating that the reactions were specific.

### Effect of silencing *Btk29A* and *COL3A1* on survival of *Thrips palmi*

*T. palmi* fitness was significantly affected post-exposure to *Btk29A* and *COL3A1* dsRNAs under controlled laboratory settings. Feeding on *Btk29A* dsRNA at a concentration of 3 μg/mL for 24 h induced 60% mortality in *T. palmi* adults (Figure 3). Whereas the mean mortality of *COL3A1* dsRNA-fed *T. palmi* was 50.9% compared to 5% in *T. palmi* fed on the diet without dsRNA. At a 95% confidence level, the mean mortality for *Btk29A* and *COL3A1* dsRNA treatments was significant, with *p*-values of 0.024 and 0.013, respectively, compared to the untreated control. There was no discernible difference in mortality between *T. palmi* treated with *BtTOB1* dsRNA and the untreated control. No immediate morphological deformities were recorded in *T. palmi* adults post-*Btk29A* and *COL3A1* dsRNA exposure (Figure 3).

### Effect of *Btk29A* and *COL3A1* silencing on groundnut bud necrosis orthotospovirus titer in *Thrips palmi*

**Figure 4:**
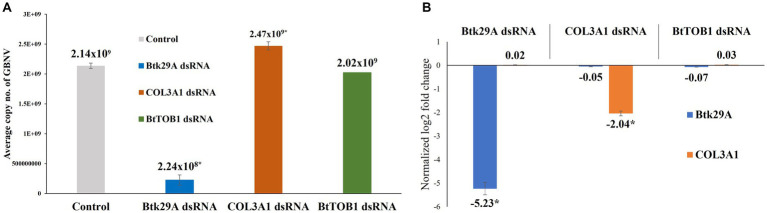
Effect of silencing *T. palmi Btk29A* and *COL3A1* on groundnut bud necrosis orthotospovirus (GBNV) titer in *T. palmi*. **(A)** Mean GBNV copy numbers acquired by *Btk29A* and *COL3A1* dsRNA-exposed *T. palmi* larvae. **(B)** Expression of *T. palmi Btk29A* and *COL3A1* mRNA in larval stage post-dsRNA exposure. The error bars are the standard error of the mean (SEM). Mean denoted by an asterisk (*) indicates a significant difference (p < 0.05).

The GBNV titer in early instar larvae of *T. palmi* was affected post-silencing *Btk29A* and *COL3A1*. Silencing of *Btk29A* induced a 9.53-fold decrease in the GBNV titer in *T. palmi*. The mean GBNV copy in *T. palmi* that were fed on the diet without dsRNA was 2.14 × 10^9^. In contrast, the mean GBNV copy acquired by *T. palmi* fed on the diet mixed with *Btk29A* dsRNA was 2.24 × 10^8^. Surprisingly, the silencing of *T. palmi COL3A1* led to a slight increase in the mean GBNV copy acquired by *T. palmi*. The mean GBNV copy in *COL3A1* dsRNA-fed *T. palmi* was 2.47 × 10^9^, i.e., 0.87-fold higher than the untreated control (Figure 4). At this stage, the expression of *T. palmi Btk29A* and *COL3A1* was downregulated by 5.23 and 2.04-fold, respectively, in dsRNA-fed *T. palmi* larvae as compared to larvae fed on the diet without dsRNA. There was no significant difference in GBNV titer following *BtTOB1* dsRNA exposure when compared to the untreated control.

## Discussion

RNAi approaches are currently less documented for members of the insect order Thysanoptera, and the function of the thrips genes in different biological and molecular processes remains unclear (Rajesh et al., 2023). The current work is the first to examine the effect of silencing *T. palmi* genes employing dsRNA. In response to GBNV acquisition, numerous *T. palmi* transcripts are linked with innate immune response, receptor binding, cell signaling, cellular trafficking, viral replication, and cellular apoptosis (Jagdale and Ghosh, 2019; Mahanta et al., 2022). Response of more than a hundred genes of *T. palmi* is conserved to GBNV and CaCV infection (Mahanta et al., 2022). The expression of *T. palmi Btk29A* and *COL3A1* were significantly regulated in response to orthotospovirus acquisition (Widana Gamage et al., 2018; Mahanta et al., 2022). In the present study, *T. palmi Btk29A* and *COL3A1* were silenced using exogenous uptake of dsRNA, and the resulting effects were assessed.

DsRNA was administered to *T. palmi* orally mixed with an artificial diet. In our previous study, a setup was optimized for *T. palmi* to deliver antisense oligos (Priti et al., 2022). The same setup was used in the current study for the oral delivery of dsRNA molecules. Oral dsRNA delivery has previously been described in *T. tabaci* and *F. occidentalis* (Jahani et al., 2018; Singh et al., 2019; Andongma et al., 2020). Different artificial diets such as Luria-Bertani and Tryptone-Soy-Broth and Yeast-based diets were used for delivery of dsRNA in *F. occidentalis* (Whitten et al., 2016; Andongma et al., 2020). However, these diets were not suitable for *T. palmi* (Priti et al., 2022). In the current research, an extract of pine pollen and sucrose was used that has been optimized for *T. palmi*. In this setup, *T. palmi* thrives for more than 12 days. A blue dye was blended with the diet that was clear through the thrips cuticle thereby confirming the active feeding by thrips. Previously the stability of dsRNA in gut juice and hemolymph was reported in thrips species (Jahani et al., 2018). No degradation of *V-ATPase-B* dsRNA (1 μg/μl) was reported in *F. occidentalis* at 0, 1, 2, and 3 days post-oral delivery. Besides oral delivery, leaf-disk, microinjection, and plastid-mediated delivery were reported in *F. occidentalis* (Badillo-Vargas et al., 2015; Han et al., 2019; Gao et al., 2020; Wu et al., 2022).

In the present study, oral administration of dsRNAs reduced *T. palmi Btk29A* and *COL3A1* mRNA expression levels by 3.62 and 3.15-fold, respectively, at 24 h. In *T. palmi,* dsRNA targeting *B. tabaci TOB1* (nonspecific to *T. palmi*) was used as a negative control in *T. palmi*. There was no significant alteration in the expression of *T. palmi Btk29A* and *COL3A1* post-*BtTOB1* dsRNA treatment. Further, there was no significant difference in the expression of the endogenous control gene, *β-tubulin* between dsRNA-exposed and non-exposed *T. palmi*, indicating that the dsRNA had a specific effect on the target genes. In previous reports, a 25% decrease in the *V-ATPase-B* mRNA levels was reported in *F. occidentalis* 3 days post microinjection of *V-ATPase-B* dsRNA (Badillo-Vargas et al., 2015). Silencing of *F. occidentalis TLR6*, *apoLp*, *COPE*, *SAMM50,* and *hsp11.6* was also achieved using dsRNA (Han et al., 2019; Yuan et al., 2022). In *T. tabaci*, oral delivery of *SNF7* and *AQP* dsRNAs caused 16.4 and 14.47-fold reductions in respective mRNA levels (Singh et al., 2019). However, the downregulation of *T. palmi* mRNA in the present study was relatively lower as compared to other thrips species reported previously. The dsRNA concentration, exposure time, target gene expression, delivery method, thrips species, and the defense mechanism of the host might be the reasons behind the variation in the magnitude of silencing (Ramkumar et al., 2021). Silencing of *T. palmi Btk29A* and *COL3A1* negatively influenced had a detrimental impact on *T. palmi* fitness. Adult *T. palmi* showed up to 60% mortality 24 h post-dsRNA exposure when compared to *T. palmi* fed a diet without dsRNA and a diet containing *BtTOB1* dsRNA. *Btk29A* and *COL3A1* are involved in a series of molecular and biological processes in *T. palmi* and depletion of *Btk29A* and *COL3A1* mRNA might interrupt the essential processes for the survival of *T. palmi*. Increased mortality and decreased fertility were previously reported in *V-ATPase-B* dsRNA-injected *F. occidentalis* (Badillo-Vargas et al., 2015). Silencing of *α-tubulin* also caused high mortality in *F. occidentalis* larvae (Whitten et al., 2016). High mortality was also reported in adults of *F. occidentalis* post-silencing of *TLR6*, *apoLp,* and *COPE* (Han et al., 2019). In *T. tabaci*, up to 72% mortality was reported post-silencing of *SNF7* and *AQP* (Singh et al., 2019). Besides the dsRNA-mediated mRNA depletion, disruption of *T. palmi UHRF1BP1* and *PFAS* expression using modified antisense oligos resulted in 93.33% mortality. Morphological deformities were recorded post-silencing of *UHRF1BP1* and *PFAS* (Priti et al., 2022). However, in the present study, no such morphological deformities were recorded in adults of *T. palmi* 24 h post-oral delivery. Either the exposure was too short to produce any obvious immediate effects on the structural shape or *Btk29A* and *COL3A1* might not be associated with morphogenesis of *T. palmi* at all.

Silencing of *T. palmi Btk29A* and *COL3A1* also altered the orthotospovirus titer in *T. palmi* larvae. Orthotospoviruses are transmitted by *T. palmi* in a persistent-propagative mode (Ghosh et al., 2017, 2019). It is known that the virus can only be acquired by the early instar larvae, with the emerging adults becoming virulent (Ghosh et al., 2021). Therefore, the orthotospovirus titer in *T. palmi* was assessed in the larval stage. Silencing of *T. palmi Btk29A* reduced the virus copies in larval *T. palmi* by 9.53-fold. *Btk29A* belongs to the non-receptor tyrosine kinase family that participates in biological processes such as protein tyrosine kinase activity, ATP binding, protein phosphorylation, and intracellular signal transduction (Kumar et al., 2011). In human lung epithelial cells, *Btk29A* participates in host cell receptor tyrosine kinase signaling to promote influenza virus RNA synthesis, viral ribonucleoprotein (vRNP) nuclear export, and virus release. Other tyrosine kinases have also been found to be associated with virus replication in humans (Kumar et al., 2011). Inhibiting Btk kinase activity induces HIV-1-infected HLM-1 cells to undergo selective apoptosis (Guendel et al., 2015). The decrease of GBNV copies post-silencing of *T. palmi Btk29A* strongly suggests its involvement in virus replication in insect cells. In contrast, silencing of *T. palmi COL3A1* induced a small but significant increase in GBNV copies in larval *T. palmi*. *COL3A1* is associated with the innate immune response against several diseases in humans (Thomas et al., 2007; Kuivaniemi and Tromp, 2019). Collagen is also involved in transfer of viral protein from infected to acceptor cells (Millen et al., 2019). Besides cell adhesion, migration, type III collagen interacts with cell surface receptor integrins (Kim et al., 2005). However, its role in antiviral resistance has never been explored. The depletion of *COL3A1* mRNA probably aided the virus to evade the innate immune response which led to an increase in virus copies in *T. palmi*.

The current study is the first to validate that silencing *Btk29A* and *COL3A1* affects the fitness and orthotospovirus titer in *T. palmi*. *Btk29A* and *COL3A1* would be novel genetic targets for sustainable thrips management and help elucidate the thrips-orthotospovirus relationships.

## Data availability statement

The datasets presented in this study can be found in online repositories. The names of the repository/repositories and accession number(s) can be found in the article/Supplementary material.

## Ethics statement

Ethical review and approval were not required for the study on animals in accordance with the local legislation and institutional requirements.

## Author contributions

VR: Data curation, Formal analysis, Investigation, Validation, Writing – original draft. SJ: Data curation, Formal analysis, Investigation, Software, Writing – original draft. AG: Conceptualization, Funding acquisition, Methodology, Project administration, Resources, Visualization, Writing – review & editing.

## Footnotes

^1^ https://www.invivogen.com/sirna-wizard

^2^ https://blast.ncbi.nlm.nih.gov/
